# Behavioural and genetic correlates of malnutrition

**DOI:** 10.1017/S0007114526106370

**Published:** 2026-05-14

**Authors:** Eunice Nortey, Matilda Asante, Freda Intiful, Richmond Aryeetey, Colin Neil Moran, Ada L. Garcia, Charlotte M. Wright

**Affiliations:** 1 Dietetics, University of Ghana College of Health Sciences, Ghana; 2 Population, Family Planning and Reproductive Health, School of Public Health, University of Ghana College of Health Sciences, Ghana; 3 Faculty of Health Sciences and Sport, University of Stirling, UK; 4 Human Nutrition, University of Glasgow, UK

**Keywords:** Malnutrition, feeding behaviour, polygenic risk, undernutrition, genetics of undernutrition

## Abstract

Childhood undernutrition is a global public health challenge, affecting children unevenly within the same household. This study assessed the behavioural and genetic correlates of malnutrition among children aged 1–3 years in a district of the Greater Accra Region, Ghana. A cross-sectional study involving 262 child-caregiver pairs was conducted. Children were classified as wasted, stunted or healthy based on anthropometric indices. Feeding behaviours – including appetite, food refusal, force feeding and maternal feeding anxiety, were assessed using the International Complementary Feeding Evaluation Tool. Saliva samples were used to genotype nine SNP associated with appetite and energy regulation and a polygenic risk score (PGRS) was generated. Wasted children had significantly lower appetite z-scores (mean difference MD (CI): –0·37 (–0·65, –0·09) and higher *z*-scores for food refusal (0·30 (0·03, 0·58)) and caregiver feeding anxiety (0·67 (0·39, 0·94)) compared with healthy children. Maternal feeding anxiety attenuated the association between appetite and weight for height *z*-score while remaining a strong independent predictor. No associations were found between feeding behaviour and stunting. Although force feeding was common (33 % of children), it did not differ by nutritional status. The SNP rs2274333 showed a higher frequency of homozygosity for the AA genotype in wasted children. The PGRS was significantly associated with low appetite (*p* = 0·046) but not with food refusal or nutritional status. Children with wasting had a lower appetite and a higher food refusal. This is associated with high levels of maternal feeding anxiety, but does not seem to have a strong genetic basis.

Optimal childhood nutrition is essential for growth, development and long-term health^(1,2)^, and childhood is a pivotal period for developing food habits that may persist into adulthood^(3)^. Addressing malnutrition in young children is thus crucial for long-term health and aligns with the United Nations Sustainable Development Goal 2: ‘Zero Hunger’^(4)^. Despite progress, malnutrition in all its forms (undernutrition, overnutrition and micronutrient deficiencies) in the first 1000 days remains a pressing global health concern, particularly in low- and middle-income countries. The WHO recommends that children should consume a minimum of five out of eight food groups daily for adequate nutrient intake^(5)^ but only one-third of children aged 6–23 months globally achieve the recommended dietary diversity, with lower rates among younger children^(6)^. In Ghana, only 41 % of children meet this threshold while stunting (18 %) and wasting (6 %) remain prevalent^(7)^.

However, not all children exposed to similar adverse environments are undernourished, and responses to nutritional interventions vary^(8–10)^. This suggests that individual-level factors, including behavioural and genetic influences, may be important determinants of nutritional outcomes. Feeding practices and child eating behaviours also matter. Responsive feeding which requires attention to children’s hunger and satiety cues promotes healthier appetite regulation, whereas overly controlling or laissez-faire feeding styles may undermine eating behaviours^(11–14)^.

Beyond caregiving, children’s genetic predispositions may shape appetite and energy regulation^(15)^, with traits such as satiety responsiveness and sensitivity to food cues being highly heritable^(16,17)^. While much attention has focused on genetic risk for obesity, evidence also suggests a role for genetics in thinness and undernutrition^(16)^. The interaction between feeding behaviours and genetic predispositions may therefore help our understanding of why some children thrive despite adversity, while others develop malnutrition.

Therefore, this study aimed to investigate the behavioural and genetic correlates of malnutrition among Ghanaian children, offering a nuanced perspective that can inform more targeted and effective interventions. Specifically, it investigated whether wasted children exhibit lower appetite and higher food refusal compared with their stunted and well-nourished peers and explored the contribution of selected SNP associated with appetite regulation and thinness. We hypothesised that wasted children will demonstrate lower appetite and higher food refusal than stunted or well-nourished children and that genetic variants related to appetite regulation and thinness will be associated with nutritional status.

## Methods

### Study design, setting and participants

This cross-sectional study was conducted in the Ashiedu Keteke Municipal District in the Greater Accra Region, Ghana, focusing on children aged 1–3 years. The Ashiedu Keteke sub-metropolitan district, the smallest in Accra, is a bustling economic hub that attracts over 2 million people daily, putting pressure on infrastructure and sanitation. As one of the first districts to host community-based management of acute malnutrition (CMAM) learning sites, it remains a major hotspot for malnutrition, covering key slum areas in Accra.

The study targeted children within this age range who attended child welfare clinics in the district, accompanied by their primary caregivers who were directly involved in feeding and cooking for the child. This caregiver involvement ensured reliable data on feeding practices and dietary patterns. Ethical approval was granted by the Ethics Review Committee of the Ghana Health Service (GHS-ERC:022/11/21).

### Sample size and sampling procedure

Using Altmann’s normogram^(18)^, we concluded that a sample size of approximately 250 with equal numbers of wasted and normal children would provide 80 % power to detect a difference in genetic score of 1/3 sd. To obtain the desired sample, purposive sampling was used only to select specific communities and child welfare clinics where a sufficient number of malnourished children could be accessed. Within the clinics, healthy children were selected using systematic sampling with a random start, while all eligible malnourished children attending the clinic during the data collection period were invited to participate. This process was repeated at each site until the required sample sizes for both healthy and malnourished children were reached.

### Data collection procedure

Caregivers were interviewed using the International Complementary Feeding Evaluation Tool questionnaire, a structured, interviewer-administered instrument that captures key aspects of child/caregiver feeding behaviour, including appetite, food refusal, force feeding and caregiver feeding anxiety. This tool was developed specifically for use in low- and middle-income countries and validated across various languages and regions to allow it to be used in diverse settings^(19)^. The interview schedule was printed in English, but interviews were conducted in English or in Twi and Ga, the predominant languages in the study areas, to ensure comprehension and comfort for the caregivers. The researchers were trained in the use of the tool, and it was pretested among 10 Ghanaian caregivers to ensure comprehension and contextual relevance before data collection. Each interview took approximately 30 min to complete, providing an in-depth assessment of each child’s feeding behaviour and nutritional context. Data collection was carried out from April 2022 to December 2022.

### Assessment of eating behaviour

The International Complementary Feeding Evaluation Tool questionnaire includes questions on meal frequency and self-feeding. It uses a 5-point scale to evaluate specific feeding behaviours: enthusiasm for eating (appetite), food refusal, force-feeding practices and caregiver feeding anxiety.

### Dietary assessment and dietary diversity

A 24-hour dietary recall was conducted, where caregivers were asked to list all foods and drinks consumed in the 24 h prior to the time of assessment, following standard dietary assessment procedures^(20)^. The researcher, a dietician, trained her research assistants to use probing and reiterated responses to ensure completeness.

Further, a FFQ embedded within the International Complementary Feeding Evaluation Tool questionnaire was used to determine the frequency of consumption from ten food groups including starchy foods, meat/fish/poultry, dairy products, legumes/nuts, fruits, leafy vegetables, savoury snacks, sweet snacks and food cooked in oil. Caregivers rated the frequency of foods as being consumed by the child more than once daily, once daily, once a week but not daily, once a month but not weekly or never/rarely.

### Anthropometric measurements

Children’s weight and length/height were measured according to standard protocols^(21,22)^. Duplicate measurements were taken for accuracy, and the average was recorded. For children able to stand, weight was measured using a Seca 354 scale, with minimal clothing and no shoes. Those unable to stand were weighed using a baby scale, either seated or lying in the scale tray, or in a caregiver’s arms if uncooperative, with the caregiver’s weight tared. Weights were recorded to the nearest 0·1 kg.

Height was measured with a Seca 213 stadiometer for standing children, ensuring they were barefoot and in the Frankfurt plane position. For non-standing children, recumbent length was measured on a Seca 210 measuring mat. The child lay flat with gentle support from the caregiver, and measurements were recorded to the nearest 0·1 cm.

### Determination of household hunger score and wealth index

The Household Hunger Scale (HHS) was used to assess food poverty within the study population, serving as a proxy for food security levels^(23)^. This simple, culturally adaptable tool is particularly useful in regions with notable food insecurity. Caregivers responded to three key questions on food availability, hunger and instances of going without food, rating their experiences on a 4-point Likert scale from ‘0’ (never) to ‘3’ (often, more than 10 times in the past 30 days).

A wealth index was generated, based on questions about asset ownership and related variables, used in the Ghana Demographic Health Survey, and is particularly valuable in assessing economic status in developing countries^(16,24)^.

### Genetic sample collection and genotyping

#### Selection of SNP for analysis

Nine SNP associated with appetite and energy regulation were selected for analysis. Due to the under-representation of Ghanaian populations in genome-wide association studies (GWAS), SNP selection relied primarily on findings from non-African populations. Most of the SNP analysed in this study were initially identified in European and other non-African populations, with limited data on their penetrance in Ghana.

The estimated prevalences of these SNP in the British population were obtained from the Ensembl database (version 104)^(25)^. Seven of the selected SNP (rs6567160, rs10182181, rs1558902, rs13021737, rs13078960, rs543874 and rs1516725) were previously associated with thinness in a GWAS of UK adults of European descent^(16)^. These variants were considered common enough to exert a potential population-level effect. Additionally, two candidate SNP (rs2274333 and rs10246939) were selected due to their known involvement in taste perception, which may influence feeding behaviour^(26)^. This selection approach acknowledges the limitations of GWAS representation for African populations while leveraging existing genetic findings to explore potential associations with malnutrition in Ghanaian children.

### Sample collection, transport, and storage

Saliva samples for genetic analysis were collected using the ORAcollect for Paediatrics (OC-175) DNA collection kit (DNA Genotek Inc., Canada), designed for simple and reliable collection. This paediatric-friendly kit includes a swab for saliva collection from children’s mouths and contains a stabilising reagent that inhibits bacterial growth from collection through to processing. The kit is engineered to maintain DNA stability at ambient temperatures (15–25°C, up to 30°C) for up to one year.

After collection, saliva samples were transported from the study sites to the laboratory in insulated cooler bags. Upon arrival, samples were stored at room temperature on the laboratory bench until DNA extraction. Extracted DNA samples were then stored in a laboratory freezer at –20 °C until they were ready for analysis.

### DNA extraction and genotyping

DNA extraction was carried out using the DNA Genotek Oragene kit, following manufacturer’s standard protocols. DNA concentration and quality were assessed using the NanoDrop spectrophotometer (Thermo Fisher, Switzerland), evaluating the A260/A280 and A260/A230 ratios to ensure sample integrity. Genotyping was performed using TaqMan PCR, which allowed for high specificity in SNP detection.

Genotyping and polygenic risk scores (PGRS) were calculated for specific SNP associated with energy regulation, chosen due to their known roles in metabolic pathways^(16,27,28)^. The selected SNP and their nearest respective candidate genes were as follows: MC4R (rs6567160), ADCY3 (rs10182181), FTO (rs1558902), TMEM18 (rs13021737), SEC16B (rs543874), ETV5 (rs1516725), CADM2 (rs13078960), CA6 (Gustin) (rs2274333) and TAS2R38 (rs10246939).

### Statistical analysis

All data were entered and analysed using SPSS version 22. Data cleaning was performed using descriptive analyses including frequencies to identify any missing or incorrectly coded values. Discrepancies were resolved by referencing the completed questionnaires.

The outcome of interest was the child’s nutritional status (categorised as wasting, stunting, or normal weight), while the key risk factors of interest were the eating behaviour scores, dietary diversity, the PGRS, the wealth score and food poverty.

Feeding behaviour survey responses were grouped into appetite, food refusal, forced feeding and maternal anxiety scores, by summing the responses to relevant questions and expressed as z-scores derived from healthy UK infants. The scores were then categorised into low, moderate and high occurrence (Table 1).

**Table 1.** Child and caregiver feeding behaviour scores

Scores	Components	Score range	Score interpretation	*N*	%
Appetite score	Likes food Interested in food Enjoys food Enjoys eating variety Eats quickly Finishes meals	6 to 30	6–14 = low	96	36·8
			15–22 = moderate	81	31·0
			23–30 = high	84	32·2
Refusal score	Turns away Pushes food Cries Spits out Meals last for 1 h	5 to 25	5–11 = low	101	38·7
			12–18 = moderate	76	29·1
			19–25 = high	84	32·2
Force feeding score	Restrains Pours food in mouth Forcefully open mouth	5 to 15	5–8 = low	158	60·5
			9–11 = moderate	18	6·9
			12–15 = high	85	32·6
Anxiety score	Child not eating enough child’s feeding causes significant anxiety child’s variety of foods child’s behaviour at mealtimes child’s lack of interest and/or enjoyment of food child’s eating speed	6 to 30	6–14 = low	88	33·7
			15–22 = moderate	91	34.9
			23–30 = high	82	31.4

Scores assigned to each response **–** Not at all = 1, All the time = 5.

Dietary diversity was determined using the data collected from a 24-hour recall. Foods consumed were classified under the following groups: starchy foods (grains, roots and tubers), meat/fish/poultry, eggs, dairy products, legumes /nuts, vitamin A-rich fruits and vegetables, other fruits and vegetables, and breast milk. Minimum dietary diversity was considered to be 5 out of 8 food groups for children 12–23 months and 4 out of 7 food groups for children 24–36 months^(29,30)^.

Anthropometric data were converted into z-scores using the WHO Anthro (plus) survey analyser. Using the WHO cut-offs, children were categorised as healthy (WHZ > –2 sd and/ or HAZ > –2 sd, wasted (weight for height *z*-score (WHZ) < –2 sd), stunted (height for age *z*-score (HAZ) < –2sd) and underweight (weight for age *z*-score (WAZ) < –2 sd)^(31)^.

PGRS: A simple PGRS was calculated by summing the risk alleles for each child. A score of 1 was assigned for heterozygote risk alleles and a score of 2 for homozygote risk alleles. The highest possible score was 18, assuming the child was homozygote for all risk alleles across the 9 SNP tested. To assess the genotypic distribution of the SNP, Hardy–Weinberg equilibrium was tested for each SNP using the *χ*^2^ test. SNP with p values greater than 0·05 were considered to be in equilibrium, indicating no significant deviation from the expected allele frequencies in the population. All genotyped SNP passed the Hardy–Weinberg equilibrium test, with *p* values exceeding 0·05, suggesting that the samples were representative and not influenced by genotyping errors or population stratification. Additionally, the minor allele frequencies of the SNP were compared with those from African populations with close ancestry to Ghana (i.e., Nigeria and Sierra Leone) and were found to be within the expected ranges.

A Shapiro–Wilk test was conducted to check data normality. Descriptive statistics, including means and frequencies, were used to summarise the background characteristics of children and caregivers, including their nutritional, dietary and socioeconomic factors. *χ*^2^ tests were used to assess associations between nutritional status and dietary characteristics as well as sociodemographic factors. *T* tests and multivariable linear regression examined the relationships between feeding behaviours (such as appetite, food refusal and force feeding) and nutritional status. Additionally, *χ*^2^ tests were used to explore the link between PGRS and both nutritional status and feeding behaviours. A *p* value < 0·05 was considered statistically significant.

## Results

The study included 262 child-caregiver pairs, of whom 75 were wasted, 53 stunted and 134 healthy. The wasted and stunted children were younger on average than the healthy children (healthy mean (sd) 20·9 (7·3) months, wasted 16·8 (5·0), stunted (18·7 (6·0); p ANOVA < 0·001)). About half of the children (46·7 %) achieved minimum dietary diversity, but wasted (60·0 %) and stunted (62·3 %) children were less likely to meet this standard. Meal frequency adherence was low, with no significant differences across nutritional groups (Table 2).

**Table 2.** Demographic and feeding characteristics of children and caregivers (*N* 262)

	Healthy (*n* 134)		Wasted (*n* 75)		*P χ*^2^ wasted *v*. healthy	Stunted (*n* 53)		*P χ*^2^ Stunted *v*. healthy
Characteristics	*N*	%	*N*	%		*N*	%	
**Age categories, (months)**								
12–18	64	47·8	53	70·5	**<0·001**	32	60·4	0·116
19–24	31	23·1	15	20·0		11	20·8	
>24	39	29·1	7	9·3		10	18·9	
**Sex**								
Male	57	42·5	34	45·3	0·696	25	47·2	0·656
Female	77	57·5	41	54·7		28	52·8	
**Minimum dietary diversity score (*****N*** **261)**								
Yes	72	54·1	30	40·0	0·051	20	37·7	
No	61	45·9	45	60·0		33	62·3	0·056
**Caregiver**								
Mother	122	91·0	70	93·3	0·556	48	90·6	0·889
Other	12	9·0	5	6·7		5	9·4	
**Education level completed**								
No school-primary	25	18·7	16	21·3		14	26·4	**0·029**
Junior high school	46	34·3	40	53·3		25	47·2	
Senior high/vocational/technical	42	31·3	11	14·7	**0·032**	9	17·0	
Tertiary	21	15·7	8	10·7		5	9·4	
**Marital status (*N* 261)**								
Married/consensual union	118	88·7	67	89·3	0·939	49	92·5	0·358
Separated/divorced/widowed	8	6·0	4	5·3		3	5·7	
Never married	7	5·3	4	5·3		1	1·9	
**Employment status**								
Employed	109	81·3	58	77·3	0·489	38	71·7	0·215
Unemployed	25	18·7	17	22·7		15	28·3	
**Food poverty**								
Food poor	17	12·7	5	6·7	0·175	6	11·3	0·831
Not food poor	117	87·3	70	93·3		47	88·7	
**Wealth quintile (*N* 260)**								
Poor	16	11·9	17	23·3	**0·004**	19	35·8	**<0·001**
Lowest middle	20	14·9	19	26·0		12	22·6	
Middle	34	25·4	13	17·8		6	11·3	
Upper middle	35	26·1	14	19·2		10	18·9	
Richest	29	21·6	10	13·7		6	11·3	

*p* < 0·05 – significant, Minimum dietary diversity – 5 out of 8 food groups for children 12–23 months and 4 out of 7 food groups for children 24–36 months, healthy – weight for height *z*-score (WHZ) > –2 sd and/or height for age z-score (HAZ) > –2 sd, wasted WHZ < –2 sd, stunted – HAZ < –2 sd, Food poverty measured as household hunger score.

Most caregivers were mothers (91·6 %); those of stunted children were slightly younger, while those of wasted children were slightly older than caregivers of healthy children: healthy – mean (sd) 31·6 (8·9) years, wasted 32·3 (9·3) stunted (28·2 (7·1); *P* ANOVA = 0·025. Caregivers of wasted and stunted children tended to have lower education levels.

Nutritional status significantly correlated with wealth quintile, with wasted and stunted children concentrated in poorer quintiles. Food insecurity, assessed using the HHS, was present in 10·7 % of households. This measure captures only severe household hunger, which explains the lower prevalence compared with national FAO estimates that include both moderate and severe food insecurity.

Children with wasting exhibited poorer feeding behaviours compared with both stunted and healthy children, with nearly half of caregivers reporting that wasted children had low appetite and higher rates of food refusal (Table 3). These effects were independent of age and wealth quintile.

**Table 3.** Association between feeding behaviour and nutritional status of children (t test) (*N* 262)

Feeding behaviour Nutritional status	Appetite *z*-score		*p* value	Food refusal *z*-score		*p* value	Force-feeding *z*-score		*p* value	Feeding anxiety *z*-score		*p* value
	Mean	sd		Mean	sd		Mean	sd		Mean	sd	
**Wasted**	**–0·27**	**1·1**	**0·01**	**0·22**	**1·1**	**0·032**	**0·11**	**1·1**	**0·168**	**0·49**	**1·0**	**<0·001**
**Healthy**	**0·10**	**0·9**		**–0·08**	**0·9**		**–0·08**	**0·9**		**–0·18**	**1·0**	
Mean difference (CI)	–0·37	–0·65, −0·09		0·30	0·03, 0·58		0·19	–0·08, 0·47		0·67	0·39, 0·94	
Stunted	0·13	1·0	0·844	–0·10	1·1	0·93	0·05	1·1	0·418	–0·21	1·0	0·872
Healthy	0·10	0·9		–0·08	0·9		–0·08	0·9		–0·18	1·0	
Mean difference (CI)	0·03	–0·28, 0·34		–0·01	–0·31, 0·29		0·13	–0·18, 0·44		–0·02	–0·33, 0·28	
Wasted	–0·27	1·1	**0·036**	0·22	1·1	0·11	0·11	1·1	0·745	0·49	1·0	**<0·001**
Stunted	0·13	1·0		–0·10	1·1		0·05	1·1		–0·21	1·0	
Mean difference (CI)	–0·40	–0·77, −0·03		0·32	–0·07, 0·71		0·06	–0·33, 0·46		0·69	0·35, 1·03	

*p* ·< 30.05 – significant, malnourished-either stunted or wasted or both. *Z*-scores represent standardised feeding behaviour scores, calculated by subtracting the mean score and dividing by the sd. Positive values indicate above-average feeding behaviour scores, while negative values indicate below-average scores. Nutritional status was classified using WHO growth standards: Stunting (HAZ < – 2 sd), Wasting (WHZ < –2 sd), and Healthy (HAZ and WHZ ≥ –2 sd).

Caregiver feeding anxiety was significantly higher for wasted, but not stunted children, compared with healthy. When maternal anxiety was added to a linear regression model with appetite as a predictor and WHZ as an outcome, the association of appetite was attenuated to non-significance (unadjusted *β* for WHZ = 0·227 *P* < 0·001, adjusted *β* = 0·067 *P* = 0·150) while maternal anxiety remained highly significant (*β* = –0·69 *P* < 0·001). The same effect was observed where food refusal was the outcome (unadjusted *β* for WHZ = –0·194 *P* = 0·002, adjusted *β* = 0·037 *P* = 0·46; maternal anxiety *β* = –0·63 *P* < 0·001). No significant associations were found between feeding behaviour and stunting.

Some force feeding was seen in 39·5 % children, with high levels in 33 % and the force-feeding z-score was inversely associated with appetite (Spearman’s *R* = –0·38) and positively with food refusal (*R* = 0·344) and feeding anxiety (0·29; all *P* < 0·001) but did not differ by nutritional status.

The genotype frequencies of participants across nutritional status groups are shown in Table 4. While most SNP showed no significant differences between healthy and malnourished children, SNP rs2274333 near the **CA6** gene was significantly associated with wasting. A higher proportion of wasted children (87·1 %) were homozygous for the effect allele (AA) compared with healthy children (76·0 %; *P* χ^2^ = 0·018).

**Table 4.** Genotype of children across different nutritional status groups Table 4 long description.

SNP	Closest gene	No. of samples	Effect allele	Genotype	Total		Healthy		Wasted		Stunted		*P* value healthy *v*. wasted	*P* value healthy *v*. stunted	*P* value wasted *v*. stunted
					*N*	%	*N*	%	*N*	%	*N*	%			
rs13021737	TMEM18	181	G	AA	3	1·7	2	2·1	1	2·3	0	0·0	0·824	0·388	0·573
				AG	24	13·3	15	15·6	5	11·6	4	9·5			
				GG	154	85·1	79	82·3	37	86·0	38	90·5			
rs10246939	TAS2R38	220	C	CC	59	26·8	26	22·2	23	36·5	11	25·0	0·117	0·932	0·442
				CT	118	53·6	66	56·4	30	47·6	24	54·5			
				TT	43	19·5	25	21·4	10	15·9	9	20·5			
rs543874	SEC16B	219	G	AA	89	40·6	46	40·7	28	44·40	15	34·9	0·887	0·773	0·587
				AG	109	49·8	56	49·6	29	46·0	24	55·8			
				GG	21	9·6	11	9·7	6	9·5	4	9·3			
rs1516725	ETV5	203	C	CC	137	67·5	70	69·3	43	71·7	24	57·1	0·78	0·374	0·252
				CT	61	30·0	29	28·7	15	25·0	17	40·5			
				TT	5	2·5	2	2·0	2	3·3	1	2·4			
rs2274333	CA6	208	A	AA	168	80·8	79	76·0	54	87·1	35	83·3	0·018	0·551	0·159
				AG	36	17·3	24	23·1	5	8·1	7	16·7			
				GG	4	1·9	1	1·0	3	4·8	0	0·0			
rs13078960	CADM2	220	G	GG	0	0·0	0	0·0	0	0·0	0	0·0	0·531	0·353	0·184
				GT	12	5·5	6	5·1	2	3·1	4	9·1			
				TT	208	94·5	111	94·9	62	96·9	40	90·9			
rs1558902	FTO	215	A	AA	1	0·5	1	0·9	0	0·0	0	0·0	0·655	0·289	0·081
				AT	18	8·4	10	8·9	7	11·7	1	2·3			
				TT	196	91·2	101	90·2	53	88·3	42	97·7			
rs6567160	MC4R	195	C	CC	10	5·1	6	6·0	2	3·6	2	5·1	0·804	0·939	0·903
				CT	66	33·8	33	33·0	19	33·9	14	35·9			
				TT	119	61·0	61	61·0	35	62·5	23	59·0			
rs10182181	ADCY3	216	G	AA	1	0·5	1	0·9	0	0·0	0	0·0	0·773	0·363	0·255
				AG	26	12·0	12	10·5	6	10·3	8	18·2			
				GG	189	87·5	101	88·6	52	89·7	36	81·8			

Of the 262 children enrolled, 227 provided saliva samples, and high-quality DNA was successfully extracted and genotyped from 220. Ninety-eight samples failed to genotype at one or more SNP (genotyping failure rate of 7 %), leaving 122 complete datasets for PGRS analysis. The mean (sd) PGRS was 9·52 (1·51) (out of a maximum 18). There was no statistically significant difference in mean PGRS values between any of the groups, although the mean difference between wasted and stunted was 0·49 points (*p* = 0·185) (wasted *N* 32 mean (sd) 9·84 (1·44), stunted *n* 34, 9·35 (1·54), healthy children *n* 56, 9·45 (1·52)). There was no association of PGRS with mean appetite or food refusal scores, but children with PGRS above median were more likely to have low appetite (Table 5).

**Table 5.** Association between feeding behaviour, nutritional status and polygenic risk score (PGRS) (*χ*^2^) (*N* 122)

Child’s feeding behaviour	PGRS			
	Low (< 9·5)		High (>9·5)	
	*N*	%	*N*	%
Appetite				
Low	13	21·0	21	35·0
Moderate	23	37·1	11	18·3
High	26	41·9	28	46·7
*P* value	**0·046**			
Food refusal				
Low	28	45·2	33	55·0
Moderate	20	18	30·0	
High	14	9	150	
*P* value	0·456			
Nutritional status				
Wasted	13	21·0	19	31·7
Stunted	18	29·0	16	26·7
Healthy	31	50·0	25	41·7
*P* value	0·244			
Total	62	50·8	60	49·2

## Discussion

Childhood malnutrition is still a critical public health issue in urban Ghana. This study set out to investigate where behavioural, environmental and genetic factors intersect and found that wasting was associated with poor appetite and higher food refusal, mediated by caregiver feeding anxiety, whereas stunted children displayed feeding behaviours similar to their healthy peers. The study did not find the hypothesised overall link between PGRS and nutritional status but did find a significant association with appetite. This study provides novel evidence that not all children with wasting exhibit low appetite and that such behaviours are not common in stunted children, suggesting that appetite dysregulation may be more specific to acute malnutrition. These findings highlight the greater influence of modifiable factors, such as feeding behaviours and caregiver practices, compared with genetic factors in shaping nutritional outcomes.

The finding that wasted children exhibited poor appetite, higher food refusal and increased caregiver feeding anxiety is consistent with earlier studies^(32)^, which linked similar patterns to caloric deficits and acute malnutrition. Conversely, stunted children in this study demonstrated feeding behaviours comparable to healthy children, suggesting that wasting is more acutely related to feeding behaviours than stunting. Chronic malnutrition (stunting) may result from earlier prolonged inadequate nutrition and poor living conditions^(33,34)^. These findings diverge from studies in Kenya and Ethiopia, where stunted children also displayed poor feeding behaviours^(32,35)^. Differences in environmental and methodological contexts may explain these discrepancies.

Caregiver feeding anxiety emerged as the mediator of the associations between appetite and food refusal with wasting. Anxiety-driven practices, such as pressuring children to eat, can worsen food refusal and feeding difficulties^(36,37)^, though it is also possible that the parents’ perceptions of their child’s behaviour were affected by their anxiety. However, given the cross-sectional nature of this study, directionality and causality cannot be inferred. Unidentified factors such as infections, parasitic infestations, micronutrient deficiencies or weakened child–mother bonding may also underlie these associations. These findings should therefore be interpreted with caution, and longitudinal studies are needed to clarify the direction and mechanisms of causation.

Force feeding was common in this study, and its association with food refusal highlights the bidirectional relationship between caregiver practices and child feeding behaviours. It is of note, however, that while force feeding was common in this cohort, it was not related to nutritional status. Previous studies emphasise the potential harm of force feeding, including heightened aversion and disrupted eating patterns^(14,38)^. These findings emphasise the need for interventions promoting responsive feeding, where caregivers respond sensitively to children’s hunger and satiety cues.

The genetic component of this study, though preliminary, provides valuable insights into potential polygenic influences on nutritional status and feeding behaviours. No significant associations were observed between PGRS and either feeding behaviour variables or nutritional status. However, a noteworthy finding was that significantly more wasted children were homozygous for the AA genotype of rs2274333 near the *CA6* gene, which influences taste perception through its effect on salivary carbonic anhydrase VI. Although this association may have occurred by chance given the multiple tests conducted (9 × 3), previous evidence suggests that individuals with the AA genotype exhibit reduced taste sensitivity and lower fungiform papillae density^(39)^, potentially affecting appetite and dietary intake. This supports a plausible link between oral sensory function and wasting, warranting further investigation.

Existing research also supports a genetic contribution to appetite and energy regulation, though evidence for direct links with nutritional outcomes in children remains limited. For instance, Monnereau et al.^^(40)^^ identified weak associations between BMI-related variants and eating behaviours^(40)^, while Winkler et al.^^(41)^^ reported stronger genetic effects on BMI in younger adults^(41)^. Such findings suggest that genetic influences on feeding behaviours and nutritional outcomes may vary by age and environment.

Overall, the findings do not support the hypothesis that undernourished Ghanaian children carry a greater burden of genetic polymorphisms associated with thinness compared with their well-nourished peers. Nevertheless, the observed association between rs2274333 and wasting, together with the suggestive relationship between PGRS and appetite, underscores the need for more comprehensive genetic studies in African populations. Larger, adequately powered GWAS are particularly needed to validate these exploratory findings and refine population-specific PGRS.

The lack of significant associations between most SNP and nutritional status likely reflects methodological limitations. Despite the original power calculation for detecting genetic score differences, the effective sample size was reduced by missing genotyping data, which limited statistical power. The limited number of SNP included in the PGRS, together with their selection from GWAS conducted in non-African populations, may have reduced their relevance to the Ghanaian context and the likelihood of detecting significant associations. The lack of such GWAS is widely acknowledged^(42)^. This challenge is compounded by the absence of local allele frequency data, which further constrains interpretation. Nevertheless, the observed associations between certain genotypes and the phenotypes of interest suggest that these relationships are biologically meaningful and warrant further exploration. Future GWAS in African populations are essential to identify additional or stronger associations and to develop African-derived PGRS that better reflect the continent’s rich genetic diversity. Finally, because recruitment was clinic-based, the findings may not be generalisable to all Ghanaian children and should therefore be interpreted with caution as this genetic study is mainly hypothesis-generating.

The findings from this study have several implications for addressing malnutrition in urban Ghana and similar settings. *Behavioural interventions*: Efforts to improve child feeding behaviours should address both child and caregiver practices. Interventions targeting caregiver anxiety, promoting responsive feeding and discouraging force feeding could mitigate food refusal and improve caloric intake. Responsive feeding practices, characterised by caregiver sensitivity to child hunger and satiety cues, have shown promise in improving nutritional outcomes^(43,44)^. These insights can be integrated into existing community-based management of acute malnutrition (CMAM) programmes by strengthening caregiver counselling and training frontline health workers to identify and address feeding-related anxiety and maladaptive practices. Embedding such behavioural support within CMAM could enhance treatment adherence and promote sustainable recovery. *Genetic research:* Further studies exploring the genetic underpinnings of malnutrition could pave the way for precision nutrition approaches. Identifying genetic risk factors associated with poor feeding behaviours or malnutrition could enable targeted interventions for high-risk populations. *Longitudinal studies:* The directionality of associations between feeding behaviours, caregiver practices and nutritional status remains unclear. Longitudinal studies are needed to disentangle these relationships and establish causal pathways.

### Conclusion

This study suggests that children with wasting tend to have lower appetite and refuse more food. While genetics may influence appetite regulation, the impact of the chosen SNP in the Ghanaian population on nutritional status appears to be minimal compared with modifiable factors such as feeding behaviour and dietary practices. Addressing malnutrition requires a multifaceted approach that combines behavioural interventions with larger genetic research. These findings contribute to the growing evidence supporting context-specific strategies to improve child health in urban Ghana and beyond.

Our findings highlight the need to distinguish between types of malnutrition when designing behavioural or therapeutic interventions and highlight the importance of studying genetic variants in African contexts where the burden of malnutrition remains high. By objectively measuring appetite and linking it to both wasting and stunting, this study provides novel insight into the behavioural phenotype of malnutrition, with important implications for targeted screening and care.

## Table 4 Long description

The table shows genotype frequencies of participants across nutritional status groups, including healthy, wasted, and stunted categories. It lists single nucleotide polymorphisms (SNPs) with their closest genes, number of samples, effect alleles, and genotype distributions. The table has 10 rows and 14 columns. Column headers include SNP, Closest gene, No. of samples, Effect allele, Genotype, Total N, Total percent, Healthy N, Healthy percent, Wasted N, Wasted percent, Stunted N, Stunted percent, P value healthy vs. wasted, P value healthy vs. stunted, and P value wasted vs. stunted. Row labels include specific SNPs and their genotypes. Notable trends include significant associations for SNP rs2274333 near the CA6 gene with wasting, where a higher proportion of wasted children are homozygous for the effect allele (AA) compared with healthy children.

Navigate back to Table 4.
